# Management of duodenal atresia associated with situs inversus abdominus: A case report

**DOI:** 10.1097/MD.0000000000021439

**Published:** 2020-07-31

**Authors:** Shuai Qiang, Meili Fan, Qingbo Cui, Zhaozhu Li, Yu Zhou, Qiang Li, Fengyong Li

**Affiliations:** a 10th Department, Plastic Surgery Hospital, Chinese Academy of Medical Sciences and Peking Union Medical College, Beijing; b Department of General Surgery, The First Affiliated Hospital of Harbin Medical University; c Department of Pediatric Surgery, The Second Affiliated Hospital of Harbin Medical University, Harbin, China.

**Keywords:** duodenal atresia, malrotation, situs inversus abdominus, volvulus

## Abstract

**Rationale:**

Duodenal atresia in association with situs inversus abdominus is extremely rare. Care should be taken when selecting appropriate surgical methods, and caution should be exercised during the surgery to avoid misdiagnosis and mistreatment. With prompt recognition of the condition, the surgical procedure should be performed in a timely manner to achieve positive results.

**Patient concerns:**

A newborn affected by situs inversus abdominus associated with duodenal atresia, midgut malrotation, and volvulus.

**Diagnosis:**

Congenital duodenal atresia with situs inversus abdominis.

**Interventions:**

Diamond-shaped duodenoduodenostomy with appendectomy was performed, with the release of Ladd band and correction of the malrotation.

**Outcomes:**

The baby boy is thriving well with no abdominal complaints at 4 years of surgical follow-up.

**Lessons:**

Although several theories are put forward to clarify this matter, the proper cause of duodenal atresia is not well defined. Clinical symptoms and examinations can assist diagnosis, the definitive cause should be ascertained by surgical approach. And the operating surgeon must be aware of the “mirror anatomy” to prevent unnecessary injuries. Additionally, long-term prognosis for duodenal atresia are very good, therefore, careful attention in postoperative management are important in such a case.

## 1 Introduction

Situs inversus causes mirror image positioning of the abdomen with a frequency of approximately 1/5000 to 1/20,000 of the normal population.^[1]^ This condition is associated with duodenal atresia (DA), with approximately 20 cases reported in the literature up to now.^[2]^ DA has been reported in 0.9 per 10,000 live births.^[3]^ Herein, we report a case of DA associated with situs inversus abdominus (SIA) to highlight the importance of identifying this condition and the significance of being aware of the “mirror anatomy” when carrying out the operation. We share our tips because of the condition's particularity, as a poor prognosis may be caused by lack of experience.

## 2 Ethical statement and consent

The institutional review board and ethic committee of Plastic Surgery Hospital, Chinese Academy of Medical Sciences, and Peking Union Medical College approved the ethical, methodologic, and protocol aspects of this investigation. We confirm that all methods in the present study were carried out in accordance with the relevant guidelines and regulations. Informed written consent was obtained from the patient's parents for publication of this case report and accompanying images.

## 3 Case presentation

**Figure 1:**
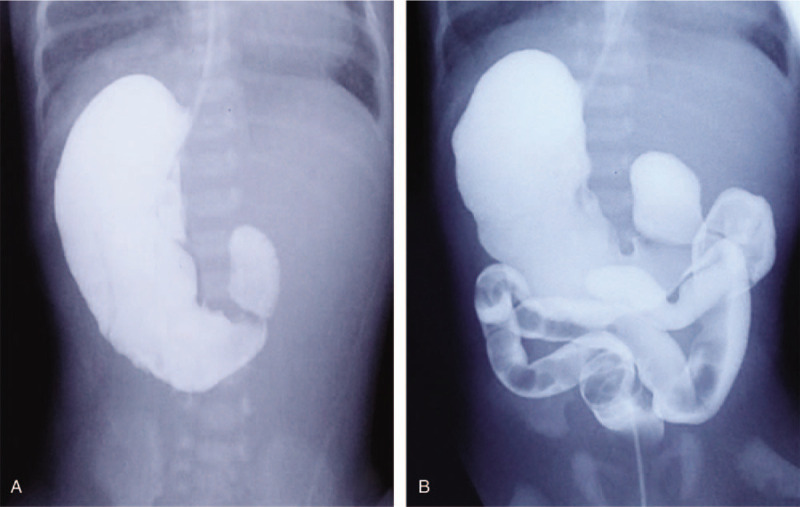
Gastroenterography was performed to evaluate the probable anatomical abnormalities. Dilated duodenal bulb and stomach were seen at 6 minutes (A), and only a little contrast medium in the colon was seen at 18 minutes (B). These images suggested the partial obstruction of the proximal duodenum.

A l-day-old boy, born weighing 3 kg by spontaneous vaginal delivery at 38 weeks’ gestation to a G3P1 mother whose pregnancy was complicated by diabetes mellitus was referred following an antenatal sonographic diagnosis of polyhydramnios and DA. The child had not passed meconium and had bile-stained vomiting. An echocardiography revealed patent ductus arteriosus (PDA) and atrial septal defect (ASD). The abdominal sonography confirmed situs inversus with the liver on the left side and the spleen on the right side. Gastroenterography was then performed to evaluate the probable anatomical abnormalities. Dilated duodenal bulb and stomach were seen at 6 minutes, and only a little contrast medium in the colon was seen at 18 minutes (Fig. 1). These images suggested the partial obstruction of the proximal duodenum.

After confirming the condition, the stomach and duodenum were decompressed, and electrolyte losses were corrected as much as possible. Surgical method: the child was placed in supine position under general anesthesia with tracheal intubation. Laparoscopic examination showed that the omentum was gathered under the liver, and the duodenum was adhered to the gallbladder. After their separation, we found that the proximal duodenum was thickened and that the distal duodenum was thinned. The junction between them presented as slightly pale and dented, which should be considered as membranous duodenal stenosis. Due to the limited operation space, we decided to transfer to open surgery to avoid secondary injuries.

**Figure 2:**
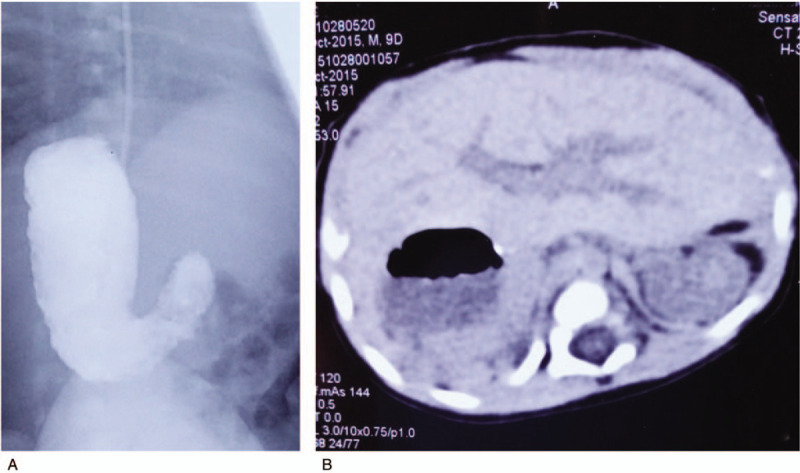
Postoperative 7th day re-examination of the upper gastrointestinal series revealed good contrast echo without extravasation (A). Computed tomography scan indicated situs inversus abdominus (B).

In the laparotomy, the liver, gallbladder, spleen, and pancreas were revealed to be situs inversus. Some of the duodenum, jejunum, and ileum were twisted 360° clockwise along the superior mesenteric artery, which presented severe congestion and edema. The gastroesophageal vessels and mesenteric vessels (veins) were highly filled, but mesenteric pulsations still existed. All of the small intestine was moved out of the incision, held in the surgeon's hands and reverted counterclockwise by 360° to the normal state. It was found that the Ladd bands constricted the surface of the duodenum and colon; therefore, a Ladd procedure was performed, and the appendix was also removed. Gastrointestinal continuity was restored by duodenal-duodenal diamond anastomosis. The infant was ventilated for 3 days postoperatively and received total parenteral nutrition for 1 week. On the postoperative 7th day, re-examination of the upper gastrointestinal series revealed that the contrast echoed well without extravasation (Fig. 2). On the postoperative 13th day, he was discharged from the hospital with cure. He remains well 4 years later.

## 4 Discussion

The SIA is characterized by inversion of the abdominal organs, with a frequency ranging from 1/4000 to 1/20,000 live births.^[4]^ SIA is commonly associated with serious cardiac and splenic malformations. Our patient had suffered from PDA and ASD without other congenital malformation. The association of SIA with DA is very rare, with no more than 30 cases reported in the literature thus far. There are 3 major types of DA: type I – these have a web formed by mucosa and submucosa with no defect in the muscle coat. A windsock deformity may occur if the web is thin. The base of the membrane is in the second part of the duodenum. Type II – duodenal ends are atretic, separated by some distance but attached by a cord. The mesentery is intact. Type III – duodenal ends are atretic, separated by some distance but without any tissue intervening. The mesentery has a V-shaped defect. Our case was diagnosed as type I, which is the most common type. However, the cause of DA is still unknown, and no specific genetic factor has been demonstrated to date. Studies indicated that DA results from the theory of “failure to recanalise” after 7 weeks’ gestation, and reported specific chromosomal anomalies or ischemic event may also play a role.^[5,6]^ The most recent research suggested that local or tissue-specific mutation in Fgf10 and Fgfr2b might represent current science's best key to unlocking this mystery.^[7]^ However, our patient did not have a chromosome check or cDNA sequencing analysis due to economic conditions. Approximately 50% of infants with DA will have polyhydramnios due to the inability to absorb amniotic fluid.^[8]^ The postnatal presentation includes obstructive symptoms, such as persistent emesis, bilious emesis, gastric distention, and/or feeding difficulties within the 1st day or 2 of life. The patient in our case presented similar symptoms when he came to our department.

To evaluate DA, perinatal ultrasound may be the 1st diagnostic test.^[9]^ Other ultrasonic signs may include a double bubble, but in our patient, it should present as “reverse double-bubble” because of SIA.^[10]^ Gastroenterography is one of the best method to locate the obstruction.^[11]^ Once DA or malrotation is identified, the patient needs surgical repair by laparotomy or laparoscopy in stable clinical condition.^[12]^ The neonates in our report still suffered from malrotation and volvulus; thus, laparotomy was performed due to the difficult visualization from the limited intraperitoneal working space. Treatment of DA with or without SIA is the same. The common options are a side-to-side or end-to-side duodenoduodenostomy or duodenojejunostomy. The survival rates and long-term prognosis for DA are very good, at approximately 90%.^[13]^ To relieve incomplete intestinal rotation, we practiced Ladd procedure, which is considered as the classic treatment for midgut malrotation, and which entailed an appendectomy.

In this case, we would like to share some of our experience regarding its management. Preoperative upper gastrointestinal angiography can identify the site of obstruction, and the double bubble sign can assist diagnosis. However, this sign is not unique to DA. Therefore, preoperative examination can only determine it; the specific cause should be ascertained through a surgical approach. This patient was suspected of DA through laparoscopy. Due to the limited operation space, we transferred to open surgery to avoid secondary injuries. It is important to determine whether there is combined intestinal necrosis while the correcting the midgut malrotation and volvulus to undertake appropriate treatment. With Ladd procedure, if the child was in good condition, the appendix can be removed to avoid a difficult diagnosis of acute appendicitis in the future. The diamond-shaped duodenoduodenostomy we adopted is a classical method for DA, which maintained the nature of the digestive tract and avoided anastomotic obstruction resulting from another approach. After the operation, care should be taken to keep the patient's temperature stable, the patient's vital signs should be monitored, and the intestinal function should be promoted. At a recent 4-year follow-up visit, our patient was developing and thriving normally.

## 5 Conclusion

In conclusion, DA in association with SIA is very rare. Although several theories are put forward to clarify this matter, the proper cause of DA is not well defined. Clinical symptoms and examinations can assist diagnosis, the definitive cause should be ascertained by surgical approach. And attention should be paid to the condition of the SIA to prevent unnecessary injuries. The operating surgeon must be aware of the “mirror anatomy” and early diagnosis. Additionally, long-term prognosis for DA are very good; therefore, careful attention in postoperative management are important in such a case.

## Acknowledgment

The authors are grateful to the patient's parents, who gave their informed consent for publication.

## Author contributions

**Conceptualization:** Meili Fan, Yu Zhou.

**Operation performer:** Meili Fan, Qingbo Cui, Zhaozhu Li.

**Writing – original draft, review & editing:** Shuai Qiang, Qiang Li,Fengyong Li.
